# Analysis of carbon emission drivers and multi-scenario projection of carbon peaks in the Yellow River Basin

**DOI:** 10.1038/s41598-023-40998-6

**Published:** 2023-08-22

**Authors:** Liangmin Wang, Weixian Xue

**Affiliations:** School of Economics and Management, Xi’an University of Technology, Xi’an, 710054 China

**Keywords:** Environmental sciences, Environmental social sciences

## Abstract

The Yellow River Basin is a key ecological barrier and commercial zone in China, as well as an essential source of energy, chemicals, raw materials, and fundamental industrial foundation, the achievement of its carbon peaking is of great significance for China’s high-quality development. Based on this, we decomposed the influencing factors of carbon dioxide emissions in the Yellow River Basin using the LMDI method and predicted the carbon peaking in the Yellow River Basin under different scenarios using the STIRPAT model. The results show that (1) the energy intensity effect, economic activity effect and population effect play a positive role in promoting carbon emissions during 2005–2020. The largest effect on carbon emissions is the population size effect, with a contribution rate of 65.6%. (2) The STIRPAT model predicts that the peak of scenarios “M–L”, “M–M” and “M–H” will occur in 2030 at the earliest. The “M–H” scenario is the best model for controlling carbon emissions while economic and social development in the Yellow River Basin. The results of this paper can provide a theoretical basis for the development of a reasonable carbon peak attainment path in the Yellow River Basin and help policy makers to develop a corresponding high-quality development path.

## Introduction

Human activities have caused and exacerbated the global climate change crisis, which will lead to a serious crisis for human society if the emission of greenhouse gases such as carbon dioxide is not strictly controlled. Carbon peaking and carbon neutrality are the keys to address climate change^1^. China has become the focus of emission reduction due to its large total and high share of carbon emissions, and its peak time and peak level have become the focus of global attention^2^. In order to cope with global climate change, in September 2020, China committed to “strive to achieve carbon peaking by 2030 and carbon neutrality by 2060”. Promoting carbon emissions to reach the peak will not only give China more voice in climate negotiations, but also drive China to achieve high-quality development. In order to help regions with conditions take the lead in achieving the peak in carbon emissions, the National 14th Five-Year Plan suggests providing support. The objective of attaining the carbon peak will ultimately be executed at the regional level because socioeconomic development in China is unevenly distributed among regions.

In addition to being a significant energy, chemical, raw material, and basic industrial base, the Yellow River Basin is also a significant location for energy consumption and air pollution in China. The Yellow River Basin’s rough development pattern of “high consumption and high pollution” has become a significant factor in delaying the achievement of the "peak carbon emission" aim. With the introduction of the Outline of the Plan for Ecological Protection and High-Quality Development of the Yellow River Basin, China has focused more on the synergy of the Yellow River Basin and emphasized the development of the Yellow River Basin as a whole. At the present stage, the energy structure of the Yellow River Basin favors coal-based fossil energy, among which three provinces, Henan, Shaanxi and Inner Mongolia, concentrate more than 70% of China's coal production, but by 2020, the power generation capacity of new energy sources such as hydro and wind power in the Yellow River Basin will only account for 26% of the entire basin. The three provinces and regions with the highest carbon emission intensity. The economic development of the Yellow River Basin is relatively lagging behind in the country, and its industrial development is dominated by the secondary industry, which in turn is dominated by primary processing, and the extraction of energy and mineral resources is common. The Yellow River Basin has a vibrant grassland pastoral industry, and its primary industry accounts for a higher percentage than the Chinese average, but the development of the tertiary industry is significantly behind the coastal areas and below the national average. Meanwhile, in recent years, the carbon emission ratio of the whole basin has reached more than one-third of the national level. The current condition of heavy industry development in the Yellow River Basin has also significantly hampered the preservation of the environment and high-quality development of the region. The Yellow River Basin has a lot of energy industries, especially coal-based industries, while in the downstream of energy, many high energy-consuming industries are derived, thermal power, building materials, iron and steel, and even coal chemical industry in turn cause pollution from the source^3^. As an important natural ecological barrier and economic zone in China, it is important to study the drivers of carbon emissions and carbon peaks in the provinces along the Yellow River Basin to achieve high-quality development in China. In this paper, we decompose the influencing factors and emission projections of carbon dioxide emissions in the Yellow River Basin.

## Literature review

The current research literature around carbon emissions mainly focuses on the analysis of carbon emission influencing factors and carbon peak prediction. For the study of carbon emission impact factors, Kaya equation^4^, structural decomposition method (SDA)^5^, exponential decomposition method (IDA)^6^, etc. are mainly used in IDA, such as Marshall-Edgeworth, Fisher, Divisia, etc. Among them, the logarithmic mean Divisa decomposition exponential method (LMDI) is the logarithmic form of Divisa exponential method.LMDI decomposition method can be further divided into additive form and multiplicative form. It has been widely valued for its advantages such as easy modeling, more desirability, more suitable for interpretation of results, and satisfying factor reversibility while eliminating residuals^7^. Therefore, this method is now widely used, for example, Kong et al.^8^ used LMDI to analyze the influencing factors of carbon emissions in China; Xu et al. ^9^ used LMDI, decomposed the carbon emissions of different provinces in China and analyzed the main influencing factors of carbon emissions in different provinces; Zhang et al.^10^ used LMDI to construct factor decomposition model of carbon emissions from background power generation, and analyzed the degree of contribution of different factors.

Carbon peak forecasting is mainly to analyze the future trend of carbon emissions, so it needs to simulate the development of economic and social factors that affect carbon emissions, etc. Scenario analysis is the most commonly used method for carbon peak forecasting. By combining scenario analysis with the LEAP model, some scholars have predicted the peak situation for China as a whole and for provinces or key regions^11–13^; some scholars have combined the STIRPAT model with the STIRPAT model to investigate the path to peak for China as a whole and for the east, central and western regions. Pathways to achieve peak^14–20^. Fang et al.^21^ combined scenario analysis with methods such as Monte Carlo analysis to calculate the emissions of eight sectors in China and predicted the carbon peaks of the eight sectors; Ma et al.^22^ analyzed the drivers of the evolution of carbon emissions of tourism in China from 2000 to 2017 and predicted the carbon peaks of tourism using scenario analysis and Monte Carlo simulation. Liu et al.^23^ decomposed the carbon emission influencing factors of industry in Henan Province and analyzed the carbon peak attainment under different scenarios; Xie et al.^24^ applied CGE to simulate how China could peak its emissions and achieve the 2 °C target by 2030 through different key countermeasures in different industries; Tian et al.^25^ used CGE mannequin to find out about the common top carbon environmental and aid influences beneath the cutting-edge countrywide goal and the trendy extra aggressive top carbon coverage in Shanghai.

As mentioned earlier, many studies predicting CO_2_ emissions have been conducted at global, national, regional and city scales. However, few studies in the literature have taken the Yellow River Basin as a whole, and in the context of China’s increasing emphasis on the overall synergistic development of the Yellow River Basin, a study of its overall CO_2_ emissions and peak carbon emissions is important for the Yellow River Basin as a whole to achieve high-quality development. The Yellow River Basin is a major concentration of solar, wind, and hydro energy resources in China, and is a renewable energy power base^26^. As one of the core basins in China, the whole basin has a heavy industrial structure, coal-fired energy structure, high energy consumption intensity, and a large proportion of “two high” industries, so it has a long way to go to promote green and low-carbon development, and therefore its carbon peaking is important for China to achieve peaking^27^. At the same time, in the previous literature, when using the STIRPAT model to analyze the carbon emissions scenarios, it is rare to divide the scenarios into positive and negative influencing factors, so that the possible scenarios can be studied more carefully and provide a stronger basis for the scenarios.

In this study, the LMDI model is used to consider the influence of drivers such as carbon emission intensity of residents, energy consumption structure, energy intensity, industrial structure, scale of economic activities, and population size on carbon dioxide emissions in the Yellow River Basin, and the extended STIRPAT model is used to determine the relationship between carbon dioxide emissions and the above drivers with the help of SPSS software package. The specific tasks of this study are (1) to decompose the carbon emissions of each province in the Yellow River Basin as well as the overall, and analyze the influencing factors of carbon emissions in each province as well as the overall; (2) to derive the amount and occurrence time corresponding to the peak carbon dioxide emissions of the Yellow River Basin as a whole under different scenarios, and put forward several policy recommendations to propose targeted carbon dioxide emission reduction measures to accelerate low-carbon development.

## Data and methodologies

### Study area and data

The Yellow River Basin flows through nine provinces and regions in Qinghai, Sichuan, Gansu, Ningxia, Inner Mongolia, Shaanxi, Shanxi, Henan and Shandong, however, only two states in Sichuan Province, Aba and Ganzi, belong to the Yellow River Basin, so this paper mainly examines the other eight provinces and regions in the Yellow River Basin.

In 2020, the Yellow River Basin will account for 30% and 25% of the country's population and GDP, respectively, and 35% and 41% of the country’s total fossil energy consumption and carbon emissions, respectively. and carbon emissions account for 35% and 41% of the country respectively. More than 3/4 of the Yellow River Basin is a moderately fragile ecological area, with soil erosion of about 260,000 square kilometers, and natural grassland degradation in the upper reaches is still relatively serious.

The total population, total GDP, GDP of three industries and urbanization rate required for the model in this paper are obtained from the statistical yearbooks of eight provinces in the Yellow River Basin from 2005 to 2020. The energy consumption of three industries (including coal, natural gas, oil, diesel, and electricity) and the proportion of non-fossil energy are obtained from the China Energy Statistical Yearbook and the China Statistical Yearbook of the Yellow River Basin for 2005–2020. Carbon emissions are based on the IPCC method, and the reference coefficients and average low-level heat generation data of various energy sources involved are based on the Chinese national standard GB/T2589-2008. The data of carbon content per unit calorific value and carbon oxidation rate used to calculate energy carbon emission coefficients are obtained from the Guidelines for the Preparation of Provincial Greenhouse Gas Inventories.

### Methodologies

#### LMDI model construction

The LMDI decomposition model has many points in common with the Kaya property, which was proposed by the Japanese scholar Yoichi Kaya^28^. The advantage of the Kaya property is the simplicity of the mathematical form and the convincing explanation of the change in carbon emissions^29^. According to Kaya;s property, the relationship between these factors can be written as:

1 $$I = \sum\limits_{ij} {I_{ij} = \sum\limits_{ij} {\frac{{I_{ij} }}{{E_{ij} }}} } \times \frac{{E_{{{\text{ij}}}} }}{{E_{i} }} \times \frac{{Q_{i} }}{Q} \times \frac{Q}{P} \times P = \sum\limits_{ij} {F_{ij} } U_{ij} S_{i} Is_{i} AP$$

where *I* denote total CO_2_ emissions, which is the amount of CO_2_ corresponding to the heat released from the combustion of fossil energy sources (mainly including coal, oil, and natural gas, etc.). *i* denotes different types of industries and *j* denotes different types of energy sources, then *I*_*ij*_ denotes the carbon dioxide produced by the *i-th* energy consumption of the *i*-th industry; *E*_*i*_ denotes the energy consumption of the *i*-th industry and *E*_*ij*_ denotes the *j*-th fuel consumption of the energy consumption of the *i*-th industry; *Q* denotes the total output level of the region, and *Q*_*i*_ denotes the gross product of the ith industry; *P* denotes the population. *F*_*ij*_ denotes the carbon emission factor, characterizing the level of technology, *U*_*ij*_ denotes the ratio of fuel *j* consumption to total consumption for industry *i*, *S*_*i*_ denotes the energy intensity of industry *I*, *I*_*si*_ denotes the ratio of GDP to GDP for industry* i*, and *A* denotes GDP per capita.

This paper chose the additive LMDI method to research energy-related CO_2_ emissions as shown in the following equations:

2 $$\Delta I_{tot} = I^{t} - I^{0} = \Delta I_{emf} + \Delta I_{mix} + \Delta I_{{\text{int}}} + \Delta I_{str} + \Delta I_{act} + \Delta I_{pop}$$

3 $$\Delta I_{emf} = \sum\limits_{i} {\frac{{I{}_{i}^{t} - I_{i}^{0} }}{{LnT_{i}^{t} - LnI_{i}^{0} }}} Ln\left( {\frac{{F_{ij}^{{\text{t}}} }}{{F_{ij}^{0} }}} \right)$$

4 $$\Delta I_{mix} = \sum\limits_{i} {\frac{{I{}_{i}^{t} - I_{i}^{0} }}{{LnT_{i}^{t} - LnI_{i}^{0} }}} Ln\left( {\frac{{U_{ij}^{{\text{t}}} }}{{U_{ij}^{0} }}} \right)$$

5 $$\Delta I_{{\text{int}}} = \sum\limits_{i} {\frac{{I{}_{i}^{t} - I_{i}^{0} }}{{LnT_{i}^{t} - LnI_{i}^{0} }}} Ln\left( {\frac{{S_{ij}^{{\text{t}}} }}{{S_{ij}^{0} }}} \right)$$

6 $$\Delta I_{str} = \sum\limits_{i} {\frac{{I{}_{i}^{t} - I_{i}^{0} }}{{LnT_{i}^{t} - LnI_{i}^{0} }}} Ln\left( {\frac{{I{\text{s}}_{ij}^{{\text{t}}} }}{{I{\text{s}}_{ij}^{0} }}} \right)$$

7 $$\Delta I_{act} = \sum\limits_{i} {\frac{{I{}_{i}^{t} - I_{i}^{0} }}{{LnT_{i}^{t} - LnI_{i}^{0} }}} Ln\left( {\frac{{A^{{\text{t}}} }}{{A^{0} }}} \right)$$

8 $$\Delta I_{pop} = \sum\limits_{i} {\frac{{I{}_{i}^{t} - I_{i}^{0} }}{{LnT_{i}^{t} - LnI_{i}^{0} }}} Ln\left( {\frac{{P^{{\text{t}}} }}{{P^{0} }}} \right)$$

where $\Delta I_{tot}$ is the total increment of carbon dioxide emissions during the study period, $\Delta I_{emf}$, $\Delta I_{mix}$,$\Delta I_{{\text{int}}}$ , $\Delta I_{str}$, $\Delta I_{act}$ and $\Delta I_{pop}$ are the influencing factors on carbon emissions, i.e. carbon emission intensity, energy consumption structure, energy intensity, industrial structure, scale of economic activities, and population size, respectively. $\Delta I_{emf}$ is the carbon emission factor utility, i.e., through technical means, it affects the carbon emission factor of energy sources, which in turn affects carbon emissions. $\Delta I_{mix}$ is the energy consumption structure effect, i.e., it affects carbon emissions by adjusting the consumption structure of energy sources. $\Delta I_{{\text{int}}}$ represents the energy intensity effect, i.e., it affects carbon emissions indirectly by adjusting energy intensity. $\Delta I_{str}$ represents the industrial structure effect, i.e., the indirect effect on carbon emissions by adjusting the industrial structure. $\Delta I_{act}$ represents the economic activity scale effect, i.e., the effect on carbon emissions when people expand their economic activities. $\Delta I_{pop}$ represents the population scale effect, i.e., the effect on carbon emissions as the population expands.

#### Modified STIRPAT model construction

Ehrlich and Holden^30^first proposed to establish the "I = PAT" constant equation reflecting the influence of socio-demographic factors on environmental stress, which relates "environmental impact (*I*)", "population size (*P*)", "wealth per capita (*A*)" and "technology level (*T*)" and is known as the environmental stress equation. Initially, the IPAT equation was widely applied to the effect of demographic factors on the environment and analyzed the problem by changing one factor while keeping the others fixed. There is some debate about I = PAT, but its biggest flaw is that it assumes the same rate of change in the elasticity of population, affluence, and technology to the environment.

To fill this gap, York^31^ constructed a stochastic STIRPAT model based on the IPAT method. The model equation can be expressed as follows:

9 $$I = aP^{b} A^{c} T^{d} e$$

where *I*, *P*, *A*, and *T* denote environmental pressure, population size, affluence, and technology level, respectively, *a* is the model coefficient, *b*, *c*, and* d* denote elasticity coefficients, and *e* is the model error term. Compared with the IPAT model, firstly, the STIRPAT model has better scalability and can introduce multiple independent variables to test the influence of each independent variable on environmental pressure when conducting environmental impact assessment; secondly, STIRPAT is a non-linear model and the introduction of exponents makes it possible to use the model to analyze the non-equal proportional influence of human factors on the environment.

To facilitate the testing of human factors on environmental of the environment and to better overcome the heteroscedasticity of the model, we take the logarithmic transformation of Eq. (9) and obtain the following model:

10 $$InI = \ln a + b(\ln P) + c(\ln A) + d(\ln T) + \ln e$$

According to the previous literature review, domestic and foreign scholars have extended the model for specific research needs when conducting related studies. In this paper, we consider the urbanization rate (*Ps*) in conjunction with the LMDI model, taking into account that the rapid economic development has brought about an increasing level of urbanization^32^, and the resulting infrastructure construction and changes in people's consumption patterns have also generated a large number of carbon emissions. The formula can be extended as follows:

11 $$InI = \ln a + b(\ln P) + c(\ln A) + d(\ln T) + e(\ln Ps) + f(\ln U) + {\text{g}}\left( {\ln I{\text{s}}} \right) + \ln {\text{h}}$$

where* I* is total CO_2_ emissions; *P* denotes population size; *A* denotes GDP per capita, representing the level of economic development;* T* denotes carbon emission intensity; *P*_*S*_ denotes urbanization rate; *U* denotes the share of non-fossil energy and *I*_*s*_ denotes the share of secondary industry. is a constant term, b, c, d, e, f, and g denote the elasticity coefficients of the explanatory variables with respect to the explained variables, and h denotes the error term.

#### Simulation of CO_2_ emissions based on scenario analyses

We employ scenario analysis to determine the effects of various parameter combination values on upcoming CO_2_ emissions. In reality, population expansion, economic expansion, and increasing urbanization all contribute to rising carbon emissions. However, reducing carbon emission intensity and adjusting energy structure and industrial structure will help reduce carbon emissions. Therefore, we divided the above drivers into two groups: positive factors (including *P*, *A*, *Ps*) and negative factors (including *T*, *U*, *Is*).

The study divides the annual rates of change of positive and negative components into three levels: low, medium, and high, designated, respectively, by "L," "M," and "H." The combination of these drivers at various levels will produce eight CO_2_ emission scenarios, which are presented in Table 1 and obviously do not take into account the "L–H" scenario. This is so because it is doubtful that the level of technology and industrial structure will advance and change quickly when social and economic development is slow or stagnant. Based on the 14th Five-Year Plan, the 14th Five-Year Plan for Energy Conservation and Emission Reduction, related policies, case studies, and other relevant literature, the future rates of change of each driver at different levels are derived. The annual rate of change of each driver for 2021–2050 was chosen as the average value for five years, and the detailed definition of the annual rate of change of drivers for 8 scenarios from 2021 to 2050 is shown in Table 2.

**Table 1:** Description of 8 scenarios.

Scnearios	Positive factors				Negative factors	
	Population size	GDP per capita	Urbanization rate	Carbon emission	The share of non-fossil energy	The share of secondary industry
L–L	L	L	L	L	L	L
L–M	L	L	L	M	M	M
M–L	M	M	M	L	L	L
M–M	M	M	M	M	M	M
M–H	M	M	M	H	H	H
H–L	H	H	H	L	L	L
H–M	H	H	H	M	M	M
H–H	H	H	H	H	H	H

**Table 2:** Definition of annual variation rates of driving factors under different scenarios (%).

Scenarios	Year	Population size	GDP per capita	Urbanization rate	Carbon emission	The share of non − fossil energy	The share of secondary industry
L–L	2021–2025	− 0.2	3.50	0.8	− 2.5	8	− 1.5
	2026–2030	− 0.25	2.50	0.6	− 1.5	5	− 0.8
	2030–2040	− 0.3	1.50	0.4	− 1	3	− 0.5
	2040–2050	− 0.4	0.50	0.2	− 0.5	2	− 0.3
L–M	2021–2025	− 0.2	3.50	0.8	− 3	10	− 2
	2026–2030	− 0.25	2.50	0.6	− 2	7	− 1.3
	2030–2040	− 0.3	1.50	0.4	− 1.5	5	− 0.8
	2040–2050	− 0.4	0.50	0.2	− 1	3	− 0.5
M–L	2021–2025	− 0.15	5.50	1	− 2.5	8	− 1.5
	2026–2030	− 0.2	4.00	0.8	− 1.5	5	− 0.8
	2030–2040	− 0.25	2.50	0.6	− 1	3	− 0.5
	2040–2050	− 0.35	1.00	0.4	− 0.5	2	− 0.3
M–M	2021–2025	− 0.15	5.50	1	− 3	10	− 2
	2026–2030	− 0.25	4.00	0.8	− 2	7	− 1.3
	2030–2040	− 0.35	2.50	0.6	− 1.5	5	− 0.8
	2040–2050	− 0.5	1.00	0.4	− 1	3	− 0.5
M–H	2021–2025	− 0.15	5.50	1	− 3.5	12	− 2.5
	2026–2030	− 0.25	4.00	0.8	− 2.5	9	− 1.5
	2030–2040	− 0.35	2.50	0.6	− 2	7	− 1
	2040–2050	− 0.5	1.00	0.4	− 1.5	5	− 0.6
H–L	2021–2025	− 0.1	7.00	1.2	− 2.5	8	− 1.5
	2026–2030	− 0.15	5.00	1	− 1.5	5	− 0.8
	2030–2040	− 0.2	3.00	0.8	− 1	3	− 0.5
	2040–2050	− 0.25	2.00	0.6	− 0.5	2	− 0.3
H–M	2021–2025	− 0.1	7.00	1.2	− 3	10	− 2
	2026–2030	− 0.15	5.00	1	− 2	7	− 1.3
	2030–2040	− 0.2	3.00	0.8	− 1.5	5	− 0.8
	2040–2050	− 0.25	2.00	0.6	− 1	3	− 0.5
H–H	2021–2025	− 0.1	7.00	1.2	− 3.5	12	− 2.5
	2026–2030	− 0.15	5.00	1	− 2.5	9	− 1.5
	2030–2040	− 0.2	3.00	0.8	− 2	7	− 1
	2040–2050	− 0.25	2.00	0.6	− 1.5	5	− 0.6

## Results and analysis

### Additive LMDI decomposition of CO_2_ emissions

In order to further explore the intrinsic influence mechanism of CO_2_ emissions and measure the influence of each influence factor in the above two stages, we adopt the LMDI method to decompose the total CO_2_ emissions into six influence factors: carbon emission factor, energy consumption structure, energy intensity, industrial structure, economic activity and population. Then we obtained the variance of the factors for each province each year, and the contribution of each factor to the carbon emission of each province and the overall Yellow River Basin from 2005 to 2020 is shown below.

It can be seen from Table 3: The energy consumption structure effect, energy intensity, economic activity effect and population effect of Qinghai Province from 2005 to 2020 play a positive role in promoting carbon emissions; carbon emission coefficient and industrial structure play a negative role in influencing carbon emissions. Among them, economic activity has the largest contribution to CO_2_ emission, with the cumulative emission reaching 5,551,200 tons. The contribution to carbon emission can be calculated, the largest is the scale effect of economic activity, contributing 76%, and the other influencing factors in order of contribution are: population scale effect is 54%, energy consumption structure is 11%, industrial structure effect is 6%, economic activity effect is − 22%, and energy consumption structure effect is − 25%.

**Table 3:** Impact of different factors on carbon emissions in Qinghai Province, 2005–2020 (Unit: 10^4^ tons).

Time period	Carbon emission coefficient	Energy consumption structure	energy intensity	Industrial structure effect	Economic activity scale	Population scale	Total
2005–2006	43.13	− 17.91	− 54.67	− 66.07	29.54	16.13	− 49.85
2006–2007	31.25	89.06	− 24	25.92	43.48	15.53	181.23
2007–2008	− 92.37	− 34.58	− 32.77	− 39.68	51.07	11.69	− 136.63
2008–2009	− 34.51	− 5.3	42.28	88.19	14.59	13.68	118.93
2009–2010	34.79	− 47.28	− 28.95	− 52.19	50.31	29.64	− 13.68
2010–2011	− 13.21	− 4.31	− 20.99	− 24.88	51.27	24.72	12.6
2011–2012	32.9	19.66	− 31.06	38.6	32.08	28.98	121.17
2012–2013	73.32	− 23.99	− 12.18	− 0.12	38.12	29.06	104.21
2013–2014	− 64.39	120.52	− 69.92	32.45	55.31	37.61	111.59
2014–2015	5.06	60.11	− 21.6	18.39	− 30.91	34.69	65.74
2015–2016	− 98.14	87.84	− 49.73	68.6	44.72	35	88.3
2016–2017	− 115.46	− 26.48	271.56	− 23.84	32.33	34.24	172.35
2017–2018	− 16.95	− 27.65	293.74	− 250.88	40.21	34.53	73
2018–2019	33.69	− 28	− 42.91	59.74	25.33	32.59	80.43
2019–2020	− 4.1	− 81.04	− 176.44	− 32.16	77.66	13.77	− 202.3
Total	− 184.97	80.64	42.37	− 157.92	555.12	391.86	727.1
Proportion	− 0.25	0.11	0.06	− 0.22	0.76	0.54	1

It can be seen from Table 4: For Gansu Province, the overall carbon emission from 2005 to 2020 is reduced, and the energy consumption structure effect, industrial structure effect and economic activity effect play a positive contribution to carbon emission; the carbon emission coefficient effect, energy intensity effect and population scale effect play a negative influence on carbon emission. The contribution to each effect carbon emission can be calculated, the largest is the economic activity scale effect, which contributes 101%, and the other influencing factors in order of contribution are: energy consumption structure effect is 23%, industrial structure effect is 7%, carbon emission effect is − 51%, population scale effect is − 80%, and energy intensity effect is − 101%.

**Table 4:** Impact of different factors on carbon emissions in Gansu Province, 2005–2020 (Unit: 10^4^ tons).

Time period	Carbon emission coefficient	Energy consumption structure	Energy intensity	Industrial structure effect	Economic activity scale	Population scale	Total
2005–2006	98.92	− 41.08	93.29	− 37.55	92.2	− 18.51	187.27
2006–2007	55.44	197.32	− 47.12	− 114.52	85.76	− 23.09	153.79
2007–2008	− 48.97	69.32	− 11.86	− 21.57	62.65	− 17.73	31.85
2008–2009	− 72.59	− 11.31	49.27	46.67	71.73	− 82.33	1.45
2009–2010	− 52.52	− 10.71	− 155.35	− 27.98	15.01	− 26.84	− 258.4
2010–2011	27.1	− 4.76	− 16.91	14.12	12.46	− 30.96	1.05
2011–2012	− 56.05	39.83	− 62.79	119.96	14.03	− 18.29	36.69
2012–2013	− 43.58	45.2	− 94.16	− 0.59	141.85	− 19.91	28.81
2013–2014	− 22.89	− 30.04	− 89.87	15.61	78.96	− 14.72	− 62.95
2014–2015	9.35	21.18	− 191.47	86.45	15.97	− 109.21	− 167.72
2015–2016	− 76.43	45.53	− 143.32	80.25	15.88	− 92.15	− 170.23
2016–2017	− 97.43	− 40.92	− 60.6	− 10.93	15.32	− 11.17	− 205.72
2017–2018	− 11.54	− 48.3	− 11.63	− 111.11	14.18	− 18.9	− 187.29
2018–2019	− 61.29	− 51.13	− 14.25	27.56	14.78	− 14.28	− 98.62
2019–2020	− 7.79	− 15.87	45.14	− 15.21	65.19	− 66.85	4.62
Total	− 360.26	164.25	− 711.63	51.17	715.99	− 564.93	− 705.42
Proportion	0.51	− 0.23	1.01	− 0.07	− 1.01	0.8	1

It can be seen from Table 5: For Inner Mongolia, the overall carbon emissions are reduced from 2005 to 2020, and the energy intensity effect, industrial structure effect and population size play a positive contribution to carbon emissions; the carbon emission factor effect, energy consumption structure effect and economic activity effect play a negative effect on carbon emissions. The contributions to carbon emissions can be calculated, in descending order, as the population size effect with 124%, the energy intensity effect with 77%, the industrial structure effect with 13%, the energy structure consumption effect with − 20%, the carbon emission factor effect with − 86%, and the energy intensity effect with − 209%.

**Table 5:** Impact of different factors on carbon emissions in Inner Mongolia, 2005–2020 (Unit: 10^4^ tons).

Time period	Carbon emission coefficient	Energy consumption structure	Energy intensity	Industrial structure effect	Economic activity scale	Population scale	Total
2005–2006	− 518.04	− 18.94	61.75	− 16.97	− 311.94	39.21	− 764.95
2006–2007	266.92	5.39	250.06	− 17.89	− 151.18	57.05	410.35
2007–2008	− 28.93	− 29.88	30.05	14.57	36.48	56.27	78.55
2008–2009	− 37.88	− 3.25	16.51	− 34.53	12.63	41.77	− 4.75
2009–2010	− 50.47	− 25.67	20.61	31.60	− 17.20	49.48	8.35
2010–2011	− 69.78	38.62	15.44	91.30	− 133.75	54.62	− 3.55
2011–2012	19.79	− 29.00	22.61	42.45	− 104.15	41.42	− 6.89
2012–2013	− 70.00	26.67	20.83	− 10.82	− 152.83	36.53	− 149.63
2013–2014	− 38.90	− 12.42	13.84	− 31.40	− 106.67	28.70	− 146.85
2014–2015	57.40	− 12.07	13.74	− 10.11	− 52.96	29.05	25.05
2015–2016	− 33.06	− 54.95	16.00	83.88	− 29.04	29.20	12.03
2016–2017	− 25.75	− 50.76	26.39	− 32.18	− 13.93	37.66	− 58.57
2017–2018	− 32.13	− 16.95	− 39.48	13.30	− 26.48	42.96	− 58.76
2018–2019	42.60	12.03	− 17.90	− 11.72	25.33	38.91	89.25
2019–2020	63.96	68.22	− 42.02	− 41.82	− 77.66	72.66	43.34
Total	− 454.27	− 102.97	408.42	69.66	− 1103.36	655.49	− 527.03
Proportion	0.86	0.20	− 0.77	− 0.13	2.09	− 1.24	1.00

It can be seen from Table 6: The overall carbon emissions in Ningxia from 2005 to 2020 are increasing, and the energy intensity effect, economic activity scale effect and population scale play a positive contribution to carbon emissions; the carbon emission coefficient effect, energy consumption structure effect and industrial structure effect play a negative effect on carbon emissions. The contribution to carbon emissions can be calculated from the largest to the smallest, which is the population size effect with a contribution of 310%, the economic activity scale effect with 264%, the energy intensity effect with 175%, the carbon emission coefficient effect with − 87%, the energy consumption structure effect with − 118%, and the industrial structure effect with − 445%.

**Table 6:** Impact of different factors on carbon emissions in Ningxia, 2005–2020 (Unit: 10^4^ tons).

Time period	Carbon emission coefficient	Energy consumption structure	Energy intensity	Industrial structure effect	Economic activity scale	Population scale	Total
2005–2006	− 143.79	5.26	35.84	− 35.62	81.46	10.55	− 46.30
2006–2007	418.30	1.45	76.33	− 31.00	77.08	13.56	555.71
2007–2008	− 123.72	− 17.79	64.65	− 42.60	− 94.23	10.97	− 202.73
2008–2009	− 110.15	− 0.82	19.72	− 209.03	57.76	50.60	− 191.91
2009–2010	112.47	− 7.91	70.76	73.74	10.51	16.66	276.23
2010–2011	46.23	10.52	− 50.55	− 45.59	125.00	20.42	106.03
2011–2012	− 19.22	− 24.95	91.00	− 19.01	20.39	13.02	61.24
2012–2013	− 54.52	− 76.84	64.14	29.43	38.39	13.10	13.70
2013–2014	− 17.74	− 53.75	68.72	− 86.60	22.28	10.17	− 56.92
2014–2015	− 17.16	− 42.47	− 85.27	70.79	19.19	80.35	25.45
2015–2016	− 32.06	− 16.31	59.79	11.71	23.91	74.26	121.31
2016–2017	− 86.84	16.32	− 15.86	− 20.17	23.20	94.82	11.46
2017–2018	− 46.13	− 55.91	− 49.82	− 462.22	11.77	17.05	− 585.26
2018–2019	− 53.26	40.87	− 18.11	− 17.75	20.90	28.68	1.33
2019–2020	− 20.42	21.78	− 33.39	28.96	11.14	72.45	80.52
Total	− 147.99	− 200.56	297.96	− 754.99	448.74	526.68	169.85
Proportion	− 0.87	− 1.18	1.75	− 4.45	2.64	3.10	1.00

It can be seen from Table 7: The overall carbon emissions in Shaanxi Province from 2005 to 2020 are increasing, and the scale effect of economic activities and population size play a positive contribution to carbon emissions; the carbon emission coefficient effect, energy intensity effect, energy consumption structure effect and industrial structure effect play a negative effect on carbon emissions. The contribution to carbon emissions can be calculated from the economic scale effect, population scale effect, carbon emission coefficient effect, industrial structure utility, energy consumption structure and energy intensity effect, in descending order.

**Table 7:** Impact of different factors on carbon emissions in Shaanxi Province, 2005–2020 (Unit: 10^4^ tons).

Time period	Carbon emission coefficient	Energy consumption structure	Energy intensity	Industrial structure effect	Economic activity scale	Population scale	Total
2005–2006	17.14	12.85	− 10.74	− 106.61	33.09	12.40	468.35
2006–2007	− 27.11	− 326.67	45.18	50.38	− 21.94	13.44	413.80
2007–2008	− 54.03	5.10	250.58	30.01	8.00	16.27	647.74
2008–2009	37.05	30.78	129.04	− 130.49	− 71.61	16.26	721.68
2009–2010	46.16	20.99	− 226.11	53.33	16.50	16.79	1498.68
2010–2011	− 16.17	− 42.83	87.82	71.35	18.23	72.79	1023.54
2011–2012	− 4.84	− 80.35	396.87	160.40	17.57	56.59	202.51
2012–2013	− 9.06	16.12	546.72	− 40.10	15.69	44.48	217.16
2013–2014	23.43	− 28.76	681.09	− 54.96	41.29	59.86	− 182.69
2014–2015	− 33.28	− 29.08	607.03	350.82	26.05	49.47	269.38
2015–2016	− 41.61	− 57.16	− 317.15	− 90.11	52.70	71.60	− 497.74
2016–2017	− 170.84	39.43	− 97.29	− 69.13	10.14	72.15	− 525.14
2017–2018	− 150.02	− 10.78	375.04	− 111.83	20.80	62.52	− 43.33
2018–2019	− 13.10	21.80	484.00	− 147.62	− 64.34	30.90	609.47
2019–2020	87.96	− 53.81	268.69	349.16	13.06	26.40	− 392.86
Total	− 429.32	− 2666.46	− 6914.68	− 733.86	14,553.01	621.92	4430.58
Proportion	− 0.10	− 0.60	− 1.56	− 0.17	3.28	0.14	1.00

It can be seen from Table 8: The overall carbon emissions in Shanxi from 2005 to 2020 are increasing, except for the carbon emission coefficient which plays a negative contribution, all other effects play a positive contribution to carbon emissions. The contribution to carbon emissions can be calculated from the largest to the smallest in order of energy intensity effect, economic scale effect, population scale effect, energy consumption structure effect, industrial structure utility, and carbon emission coefficient effect.

**Table 8:** Impact of different factors on carbon emissions in Shanxi Province, 2005–2020 (Unit: 10^4^ tons).

Time period	Carbon emission coefficient	Energy consumption structure	Energy intensity	Industrial structure effect	Economic activity scale	Population scale	Total
2005–2006	− 391.97	69.56	54.67	− 70.79	36.58	91.98	− 209.98
2006–2007	201.58	29.48	24.00	− 47.76	132.12	16.59	356.01
2007–2008	− 38.59	15.47	320.77	11.27	41.19	15.18	365.28
2008–2009	88.41	200.33	42.28	15.94	39.90	83.47	470.33
2009–2010	64.65	84.06	58.95	− 78.23	31.76	16.77	177.95
2010–2011	26.61	24.27	20.99	− 52.03	42.11	148.04	209.98
2011–2012	− 68.58	77.65	31.06	44.18	37.71	54.16	176.17
2012–2013	− 19.31	− 20.99	120.18	− 57.49	98.60	20.34	141.33
2013–2014	− 47.38	− 26.58	69.92	51.30	49.15	77.99	174.39
2014–2015	− 36.07	− 35.12	210.60	330.51	81.06	− 62.42	488.57
2015–2016	− 49.92	− 19.30	490.73	− 11.08	32.06	51.94	494.43
2016–2017	− 28.87	− 63.61	− 21.56	− 12.78	86.98	146.23	106.38
2017–2018	− 28.81	− 25.27	293.74	− 63.86	18.91	78.75	273.47
2018–2019	− 66.97	− 42.08	− 20.91	48.71	33.14	52.46	4.34
2019–2020	− 8.65	− 34.18	176.44	75.21	161.77	30.41	401.00
Total	− 403.87	233.69	1871.84	183.08	923.04	821.88	3629.65
Proportion	− 0.11	0.06	0.52	0.05	0.25	0.23	1.00

It can be seen from Table 9: The overall carbon emissions in Henan from 2005 to 2020 are increasing, except for the industrial structure effect which plays a negative role in promoting carbon emissions, all other effects play a positive role in promoting carbon emissions. The contribution to carbon emissions can be calculated from the largest to the smallest in order of population size effect, energy intensity effect, carbon emission coefficient effect, economic activity size effect, energy consumption structure utility, and industrial structure effect.

**Table 9:** Impact of different factors on carbon emissions in Henan Province, 2005–2020 (Unit: 10^4^ tons).

Time period	Carbon emission coefficient	Energy consumption structure	Energy intensity	Industrial structure effect	Economic activity scale	Population scale	Total
2005–2006	543.00	− 0.77	200.13	− 195.44	20.04	1060.37	1627.34
2006–2007	363.48	0.85	501.14	− 260.43	− 21.24	904.91	1488.70
2007–2008	− 80.51	− 1.38	530.73	28.34	− 27.20	1225.76	1675.74
2008–2009	− 57.22	− 0.48	18.55	− 66.76	− 23.88	378.62	248.84
2009–2010	23.70	10.62	1594.01	− 190.51	38.99	803.02	2279.82
2010–2011	18.02	0.49	1362.58	− 39.12	15.77	736.09	2093.83
2011–2012	− 13.12	3.10	379.62	114.89	47.20	993.41	1525.09
2012–2013	− 81.31	7.48	224.43	− 110.81	59.90	708.01	807.70
2013–2014	− 26.88	− 0.56	252.09	− 21.98	− 31.74	965.73	1136.67
2014–2015	40.27	21.13	53.91	69.32	21.01	950.24	1155.89
2015–2016	− 28.65	0.56	461.06	− 21.01	54.29	456.20	922.45
2016–2017	− 120.71	16.65	637.72	61.05	34.13	970.34	1599.17
2017–2018	− 96.71	39.68	447.28	− 118.24	38.04	603.30	913.35
2018–2019	− 43.19	− 54.95	490.11	− 521.26	5.29	409.12	285.12
2019–2020	− 54.93	− 2.81	294.86	471.47	19.22	1321.70	2049.51
Total	385.24	39.60	7448.22	− 800.50	249.84	12,486.83	19,809.23
Proportion	0.02	0.00	0.38	− 0.04	0.01	0.63	1

It can be seen from Table 10: The overall carbon emissions in Shandong Province from 2005 to 2020 are increasing, the energy consumption structure effect and industrial structure effect play a negative contribution, and all other effects play a positive contribution to carbon emissions. The contribution to carbon emissions can be calculated from the largest to the smallest in order of population size effect, energy intensity effect, economic activity size effect, carbon emission factor effect, energy consumption structure utility, and industrial structure effect.

**Table 10:** Impact of different factors on carbon emissions in Shandong Province, 2005–2020 (Unit: 10^4^ tons).

Time period	Carbon emission coefficient	Energy consumption structure	Energy intensity	Industrial structure effect	Economic activity scale	Population scale	Total
2005–2006	496.67	− 1.36	40.29	− 40.16	44.93	11.10	551.47
2006–2007	126.22	1.49	74.00	− 186.00	46.14	13.91	75.76
2007–2008	− 50.66	− 2.37	11.45	− 92.17	− 42.10	13.07	− 162.79
2008–2009	− 30.77	− 0.83	11.97	− 179.11	− 42.10	71.63	− 169.21
2009–2010	41.99	− 19.44	76.90	− 60.56	10.09	122.12	171.09
2010–2011	73.11	0.84	24.67	− 25.38	51.34	118.06	242.63
2011–2012	− 32.21	4.78	38.97	− 64.58	53.52	75.90	76.37
2012–2013	− 89.58	− 11.68	42.45	46.63	25.83	81.12	94.77
2013–2014	− 40.05	− 0.87	32.84	− 12.50	55.09	60.20	94.71
2014–2015	− 15.94	1.86	− 19.39	− 24.93	− 48.45	79.32	− 27.53
2015–2016	− 23.84	0.99	40.64	− 68.91	68.50	60.74	78.13
2016–2017	− 37.29	− 30.02	70.53	− 54.41	30.40	63.31	42.52
2017–2018	− 94.17	71.43	44.17	− 39.90	41.21	56.79	79.54
2018–2019	− 80.74	− 10.19	37.09	− 19.01	29.62	798.17	754.94
2019–2020	− 101.46	− 5.19	26.26	154.05	60.82	738.21	872.69
Total	141.26	− 0.55	552.84	− 666.94	384.82	2363.66	2775.09
Proportion	0.05	0.00	0.20	− 0.24	0.14	0.85	1.00

**Figure 1:**
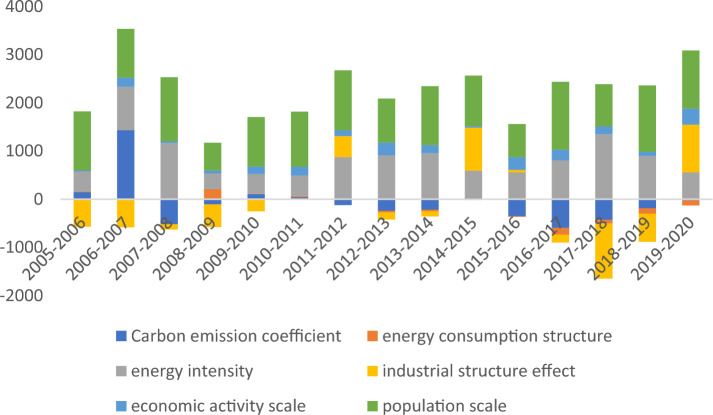
Contribution of different factors to the impact of carbon emissions in the Yellow River Basin Province, 2005–2020.

It can be seen from Table 11 and Fig. 1: The effects of various factors on CO_2_ emissions varied during the study period. Overall, it seems that the energy structure effect, economic activity effect and population effect in the Yellow River Basin from 2005 to 2020 play a positive contribution to carbon emissions; the carbon emission factor, energy consumption structure and industrial structure play a negative effect on carbon emissions. The largest contribution to carbon emissions in the Yellow River Basin is the population size effect, which contributes 60%, and the other influencing factors in order of contribution are: energy intensity effect of 41%, economic activity size effect of 8%, energy consumption structure effect of − 1%, carbon emission factor effect of − 5%, and industrial structure effect of − 6%.

**Table 11:** Impact of different factors on total carbon emissions in the Yellow River Basin, 2005–2020 (Unit: 10^4^ tons).

Time period	Carbon emission coefficient	Energy consumption structure	Energy intensity	Industrial structure effect	Economic activity scale	Population scale	Total
2005–2006	145.06	7.60	420.57	− 569.21	25.90	1223.22	1973.34
2006–2007	1436.08	− 1.63	899.59	− 581.30	190.21	1011.89	3279.34
2007–2008	− 517.79	3.90	1163.59	− 111.85	35.86	1331.48	1931.73
2008–2009	− 107.51	209.12	329.61	− 469.12	59.03	573.72	725.02
2009–2010	108.29	4.66	410.82	− 250.81	155.96	1027.64	2962.09
2010–2011	45.69	22.83	423.04	− 10.25	182.42	1143.79	2476.12
2011–2012	− 122.12	10.72	866.27	436.88	118.35	1245.19	2016.18
2012–2013	− 239.52	− 38.05	912.42	− 143.86	265.55	912.73	1116.73
2013–2014	− 217.06	− 32.46	958.72	− 108.09	163.68	1225.53	893.55
2014–2015	26.80	− 14.46	567.55	891.24	30.96	1051.52	1346.26
2015–2016	− 351.65	− 12.80	558.02	53.34	263.03	686.80	554.25
2016–2017	− 596.35	− 139.41	810.89	− 162.39	218.56	1407.58	1036.07
2017–2018	− 430.33	− 73.74	1353.04	− 1144.74	158.65	877.02	191.25
2018–2019	− 188.99	− 111.66	897.10	− 581.34	90.04	1376.54	1721.92
2019–2020	− 25.00	− 102.90	559.55	989.64	331.20	1208.75	1455.51
Total	− 1306.19	− 268.27	11,130.78	− 1761.84	2289.43	16,303.39	27,309.05
Proportion	− 0.05	− 0.01	0.41	− 0.06	0.08	0.60	1.00

It can be obtained from Fig. 1 that the population size effect, energy intensity effect, and economic size effect are all contributing to carbon emissions in the Yellow River Basin for each year from 2005 to 2020. The carbon emission coefficient effect and industrial structure effect also play a suppressive role on carbon emissions for almost every year of the study period. The energy consumption structure effect slowly shifts from being a facilitator to a suppressor of carbon emissions during the study period.

The dominant factor of increasing population size on the increase of carbon emissions in the Yellow River Basin. This conclusion is consistent with the findings of previous scholarly studies. Since the promulgation of the family planning policy, the overall population growth rate in China has been on a declining trend^33^, and the Yellow River Basin has kept pace with the national population change, but still increased by about 232,000 people per year from 2005 to 2020. The high growth in the absolute number of people is accompanied by a change in the consumption pattern of residents' life and an increase in the urban population. At this time, the growth of energy consumption is essential to ensure the survival and development of the nation and the continuous and stable growth of the economy, thus leading to a continuous rise in carbon emissions.

Energy intensity is an important factor in the increase of carbon emissions in the Yellow River Basin. Energy intensity represents the efficiency of energy economic activities, expressed in terms of energy consumption per unit GDP pair, and reflects the input versus output of the energy system^34^. The energy intensity of various industries in the Yellow River Basin continued to show an increasing trend during the study period, with a basin-wide energy intensity of 1.11 t of standard coal per million yuan in 2005 and 0.21 t of standard coal per million yuan in 2020, a figure that still has some distance to go when compared to other advanced regions in the international and domestic context, but it can be seen that the Yellow River Basin has not achieved much in terms of energy use efficiency. According to the energy intensity is related to various factors such as technology level, industrial structure and energy structure, but according to the decomposition results, the change of industrial structure and energy consumption structure contribute negatively to carbon emission, so it is concluded that the increase of energy intensity in our province is due to the lack of updating of technology level, which in turn leads to the increase of carbon emission.

The impact of the change in industrial structure on carbon emissions in this period shows a negative effect of − 6% contribution. The secondary industry always dominates carbon emissions from 2005 to 2020, with its share of emissions at 70.51% in 2005 and rising to 83.51% in 2020. In the same period, the share of carbon emissions from the primary industry increases from 3.5% to 4.2%, but the share of carbon emissions from the tertiary industry decreases from 29.01% to 16.35%. In terms of GDP, the share of primary industry GDP in 2005 was 10.17% and 8.26% by 2020, while the share of secondary industry GDP decreased from 47.51% to 43.39% and the share of tertiary industry GDP increased from 41.60% to 47.10% in the same period. The high value-added and low energy consumption industrial characteristics possessed by the tertiary industry increased the GDP share of the tertiary industry by 5.50% and reduced the proportion of carbon emissions by 12.66% during the period 2005–2020. Although carbon emissions from the secondary industry increased during the study period, carbon emissions from the tertiary industry showed a long-term decreasing trend, and its decrease offset the increase in the secondary industry. As a whole, the change in industrial structure has a negative impact on the growth of carbon emissions.

The increase in the scale of economic activities is one of the reasons for the growth of carbon emissions in the Yellow River Basin during the study period. Carbon emissions in the Yellow River Basin increased by 10% from 2005 to 2020, with a 5.28-fold increase in total GDP and a 4.84-fold increase in GDP per capita. Carbon emissions are a direct product of energy consumption, which is a basic input to keep the economic system running^35^, so carbon emissions and economic development will undoubtedly maintain a high correlation. Meanwhile, the Yellow River Basin, as a major energy province with developed industries, relies on high carbon energy consumption for economic growth, which in turn leads to a significant increase in CO_2_ emissions.

The change in the energy mix shows a strong negative effect on the growth of carbon emissions, which can be considered as the initial effect of energy mix optimization. According to the same amount of energy consumption, the larger the share of fossil energy sources, the more carbon emissions are produced by the energy system^36^. At the same time, for the same amount of fossil energy consumption, the energy type composition is different, leading to different carbon emissions due to the different carbon emission factors of each fossil energy source. Among the main fossil energy sources, the one with the smallest carbon emission factor is natural gas, which is therefore a clean energy source, followed by oil, and the one with the largest carbon emission factor is coal. Coal consumption in always dominated and its consumption scale is on a long-term upward trend. According to the data of three major energy structure changes, the tertiary sector has the biggest change, and its share of natural gas has increased by 10.88%, while its share of coal has decreased by 22.95%, so the negative effect of energy structure changes on carbon emission growth mainly comes from the adjustment of the tertiary sector, considering that the share of non-fossil energy (including hydropower, wind power, nuclear power, etc.) has increased by 6.12% in this period, which can further explain.

### The response between CO_2_ emissions and impact factors

From a macroscopic perspective, the LMDI approach can examine the primary effects and contributions of each influencing factor to CO_2_ emissions in the Yellow River basin. The strategy, meanwhile, falls short of fully capturing how carbon emissions fluctuate as an influencing factor shifts. That is, it is unable to define how changes in energy use and carbon emissions affect the drivers. Based on this flaw, we develop a better STIRPAT model to characterize the relationship between changes in CO_2_ emissions and the factors that affect them in the Yellow River Basin and to predict CO_2_ emission trends. In this work, carbon intensity is directly employed for the prediction of peak carbon emissions in the Yellow River Basin, i.e., the carbon emission factor is paired with energy intensity, in order to more intuitively characterize the amount of low-carbon technology development.

**Figure 2:**
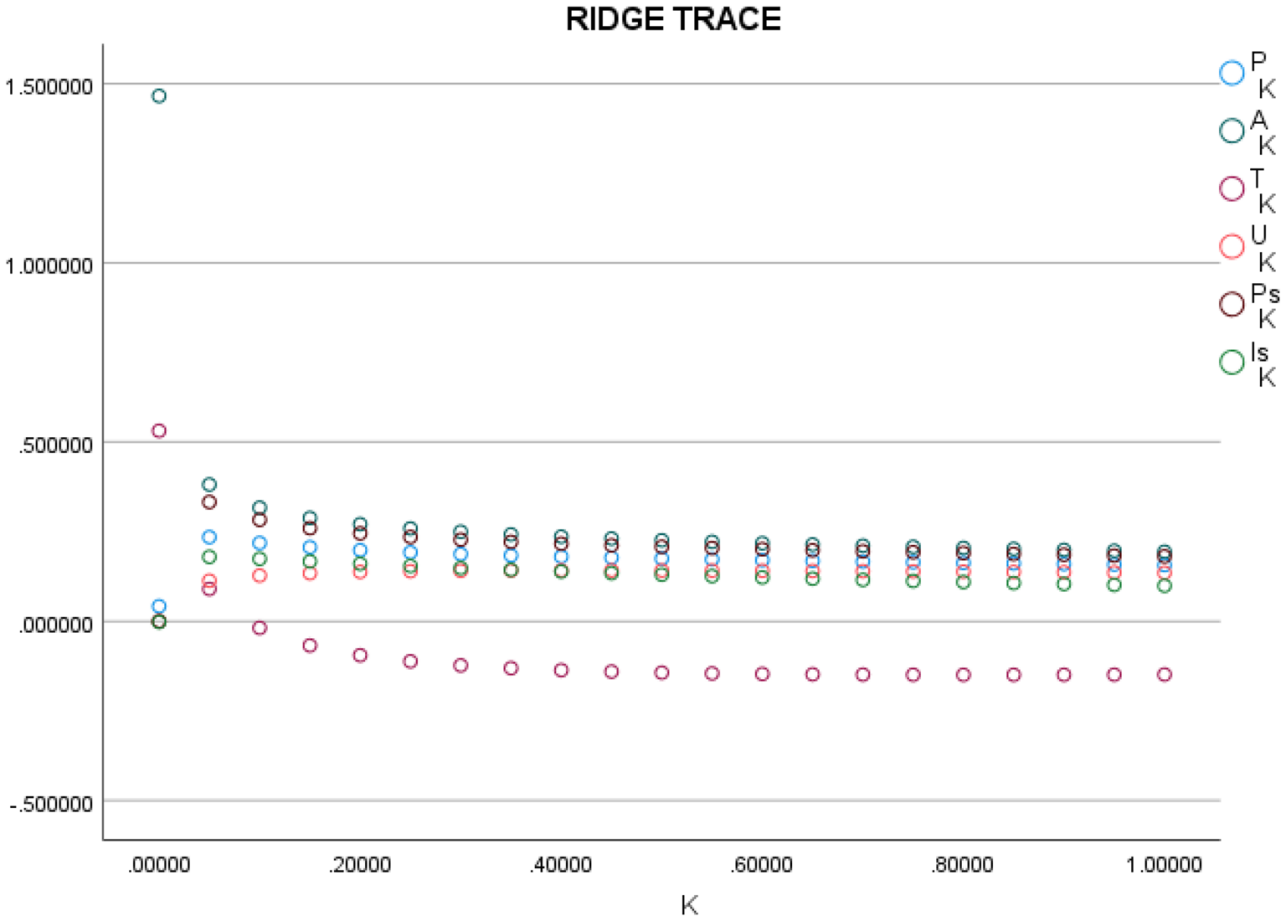
Ridge Trace Map.

**Figure 3:**
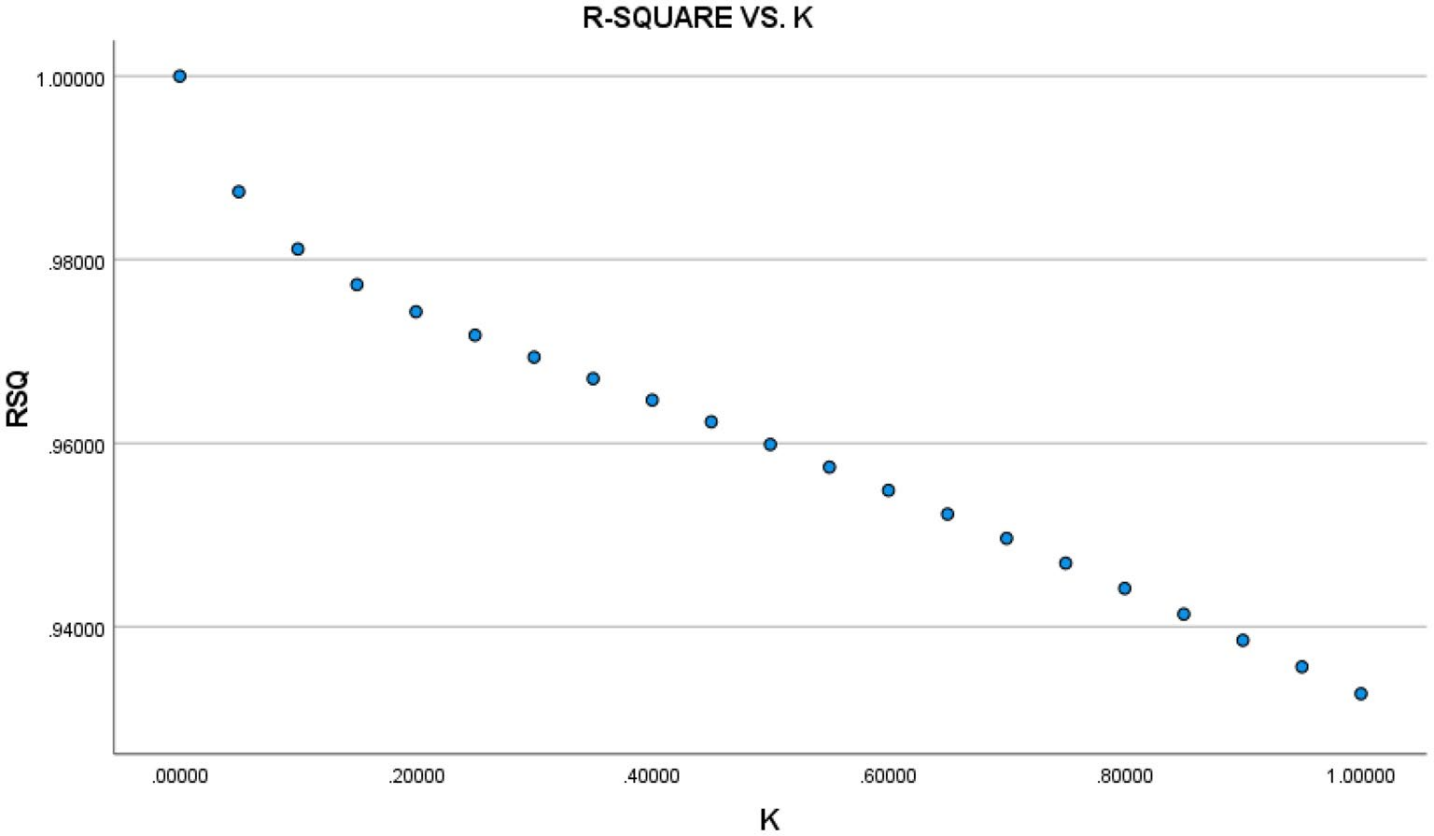
Relationship between R-squared and ridge coefficient K.

In order to eliminate the interference of multiple covariance on the experimental results, the model was analyzed and processed by the method of ridge regression in this study. Ridge regression is a biased estimation regression method specifically used to analyze covariance data. It is a more realistic and trustworthy regression method by abandoning the unbiased nature of least squares to generate regression coefficients. Hoerl and Kennard proposed the ridge regression estimation^37^. Figure 2 shows the trajectory of the ridge, and Fig. 3 shows the relationship between the resultant R-squared and the ridge coefficient K from the ridge regression.

The trend of the regression coefficients of the variables progressively stabilizes at k = 0.3, as shown in Fig. 2, where the coefficient of determination R^2^ is 0.9676. As a result, when k = 0.3, the normalized ridge regression equation is produced. Therefore, further normalized ridge regression is required to retrieve the matching unstandardized ridge regression equation in order to assess the elasticity coefficients between CO_2_ emissions and each affecting factor. Table 12's transformation results are displayed, and it is clear that every variable passes the test for statistical significance. Consequently, using the fitted parameters calculated from the ridge regression, the improved STIRPAT model can be expressed as Eq. (12).

12 $${\text{lnI}} = 0.{61} + 0.{\text{28lnp}} + 0.{\text{32lnA}} + 0.0{\text{9lnT}} - 0.0{\text{762lnU}} + 0.{\text{16lnPS}} + 0.0{\text{2lnIs}}$$

**Table 12:** Ridge regression results of CO_2_ emissions in the Yellow River Basin.

Variable	Parameter	Standard error	Standardised coefficient	t Statistics	*p*-Value
LnP	0.2826	0.0479	0.2761	5.9062	0.0035
LnA	0.3202	0.0613	0.3188	5.2241	0.0001
LnT	0.0886	0.0505	0.0869	1.7529	0.0005
LnU	− 0.0762	0.0435	0.0715	− 1.7497	0.0006
LnPs	0.1644	0.0271	0.1542	6.0563	0.0002
LnIs	0.0240	0.0527	0.0231	0.4559	0.0003
constant	0.6086	0.7159	0.0000	0.8501	0.0075
	R^2 = 0.9676		F Statistics = 14.9394	Sig.F = 0.0024	

Additionally, the CO_2_ emissions from 2011 to 2020 were obtained in accordance with Eq. (12) and the model was verified in order to further assess the extended STIRPAT model's resilience. Table 13 illustrates the contrast between the STIRPAT model's actual and anticipated values. The findings indicate that there is an average relative error of 3.44% between the predicted and actual values. This suggests that carbon dioxide emissions can be predicted using the enlarged STIRPAT model.

**Table 13:** Comparison of measured and projected CO_2_ emissions, 2011–2020.

Year	Actual values (104 tonnes)	Predicted values (104tonnes)	Relative error (%)
2011	334,143.83	345,462.33	3.39
2012	346,090.64	356,846.65	3.11
2013	359,595.84	355,484.45	1.14
2014	362,751.34	385,467.21	6.26
2015	298,282.29	318,354.51	6.73
2016	363,391.45	369,495.32	1.68
2017	370,521.01	384,563.21	3.79
2018	388,745.44	384,613.21	1.06
2019	398,849.18	378,856.32	5.01
2020	406,313.28	412,353.21	1.48

### Analysis and forecast of CO_2_ emission scenarios

It can be seen in the extended STIRPAT model that slight fluctuations in the values of these drivers may have an impact on the CO_2_ emissions and the timing of the peak carbon emissions. Therefore, this study uses scenario analysis to seek the effects of different combinations of parameter values on future CO_2_ emissions.

**Figure 4:**
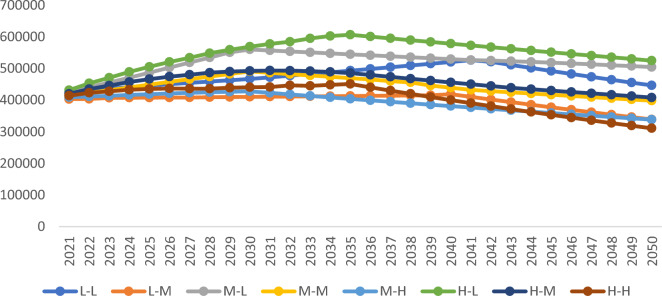
Projected CO_2_ emissions in the Yellow River Basin under 8 scenarios from 2021 to 2050.

Figure 4 shows the projected CO_2_ emissions for 8 scenarios from 2021 to 2050. Table 14 shows the peaks of the Yellow River Basin under each scenario. In terms of the time to peak, the earliest peak of scenarios “M–L”, “M–M” and “M–H” occurs in 2030, which is in line with the national target of reaching the peak in 2030, and the latest peak of scenario “L–L” occurs in 2041. L–L” scenario, which peaks in 2041. The latest scenario “L–L” peaks in 2041. "It is found that with the same growth rate of population, GDP per capita, and urbanization rate, faster technological progress, energy structure and industrial restructuring will effectively reduce the future carbon emissions in the Yellow River Basin and shorten the time to reach the peak of carbon emissions. The high rate of economic development generates carbon dioxide, which is offset to some extent by the optimization of energy structure and the development of low-carbon technology, while the progress of energy structure and low-carbon technology accelerates the pace of reaching the peak of carbon emissions. The simultaneous slow development of economic and technological progress will delay the time to reach the peak of carbon emissions.

**Table 14:** Carbon emission peak and its peak time.

Scenarios	Carbon emissions at peak	Carbon peak time
L–L	525,702.63	2041
L–M	418,391.21	2040
M–L	559,571.40	2030
M–M	490,250.10	2030
M–H	427,362.01	2030
H–L	606,075.74	2035
H–M	488,616.13	2033
H–H	450,639.36	2032

In terms of peak size, it is clear that there are significant differences in the carbon emission peaks for different scenarios, from large to small quantities, as “H–L”, “M–L”, “L–L”, “H–M”, “M–M”, “H–H”, “M–H”, and “L–M”. Taking “L–M”, “M–M” and “H–M” as examples, reducing the speed of economic and social development and maintaining the existing energy-saving and emission reduction policies can reduce the size of the peak, but cannot advance the peak time.

In practice, in order to meet the economic and social development needs of the Yellow River Basin, we should choose a scenario that can ensure the speed of economic development and achieve carbon emission reduction at the same time. The "M-H" scenario is the best model to control carbon emission while economic and social development, and it can respond to the national goal of 2030 carbon peak. To maintain a medium growth rate in this environment, socioeconomic development is required. The Yellow River Basin has recently optimized industrial structure, conducted supply-side structural reforms, and rationalized resource allocation. These changes have also changed economic growth. Since 2015, tertiary industry has dominated economic growth in the Yellow River Basin, with the share of output from secondary industry declining and the energy consumption structure progressively shifting from high-carbon coal to low-carbon natural gas. As a result, the "M-H" scenario development model satisfies the criteria for the social and economic sectors' sustainable development in the Yellow River Basin and represents a more realistic path to reaching peak carbon emissions.

## Conclusions

This study takes the Yellow River Basin as the research object, constructs a decomposition model of carbon emission influencing factors, and applies the log-average partition index (LMDI) factor decomposition method to decompose the factors influencing carbon emission changes into carbon emission coefficients, energy consumption structure, energy intensity and other influencing factors, and visually analyzes the contribution of each factor to the carbon emission changes from 2005 to 2020. Then, the extended STIRPAT model is used to explore the relationship between carbon emissions and population, GDP per capita, carbon intensity, urbanization rate, energy structure and industrial structure. Then, the extended model was fitted with ridge regression using relevant data from 2005 to 2020 using SPSS statistical software. The STIRPAT model can be used to predict future CO_2_ emissions in the Yellow River basin, according to a combination of satisfactory fitting findings and model validation. Also, this study conducted scenario analysis to determine the anticipated CO_2_ emissions in the Yellow River Basin for the period of 2021–2050 under eight scenarios, allowing researchers to explore the effects of various combinations of drivers on CO2 emissions. The results show that CO_2_ emissions peak in 2030 only under the “M–L”, “M–M”, and “M–H” scenarios, compared with the other five scenarios. The “M–H” scenario meets the requirements of sustainable socio-economic development in the Yellow River Basin. This requires socio-economic development and technological progress to maintain medium growth while continuously adjusting the energy and industrial structures.

To ensure the smooth implementation of the low-carbon development plan in the Yellow River Basin, the Yellow River Basin should make every effort to complete the control of population size within 401.3 million, maintain stable economic growth, reduce carbon emission intensity to 1.25, increase urbanization rate to 79.05%, increase the proportion of non-fossil energy to 22.34%, and reduce the proportion of secondary industry added value to GDP to 36.18% by 2030 In addition, we will adjust the economic structure, transform the mode of economic development, and strive to actively expand domestic demand. The study’s findings can help policy makers develop appropriate energy-saving and emission-reduction measures in addition to serving as a theoretical foundation for the overall construction of a peak carbon emission management framework in the Yellow River Basin and the formulation of reasonable socioeconomic development and carbon emission reduction targets.

The starting point of this paper is to jointly achieve the carbon peak target in the Yellow River Basin and to promote high-quality and synergistic development in the Yellow River Basin. Therefore, based on the results of this paper, the following suggestions are made: 1. The main source of carbon emissions in Qinghai Province is the scale of economic activities. In view of the fact that Qinghai province has the lowest carbon emissions in the Yellow River basin and its economy is relatively backward compared to other places, the main focus of Qinghai is on economic development. 2. The increase of carbon emissions in four provinces, namely Inner Mongolia, Ningxia, Henan and Shandong, is due to the expansion of population size. Shandong and Henan, as large population provinces, should control the population growth appropriately.3. For Shaanxi and Shanxi, which are large energy provinces, there are cases of successful transformation of clean coal technology and high-efficiency pulverized coal type industrial boilers, as well as the experience of successful research and development of high-efficiency equipment, so they should continue to invest in this direction and promote the efficient use of coal.4. For Gansu, the increase of carbon emissions mainly comes from economic development. Therefore, the energy supply-side reform should be promoted to make the energy-related industries develop in the direction of green and intelligent. Maximize the efficiency of energy utilization, improve the conversion capacity of natural gas, coal, oil and other energy sources, and build a high-end green energy industrial base.

## Data Availability

Materials described in the manuscript will be freely available to any researcher wishing to use them for non-commercial purposes, without breaching participant confidentiality. All authors can provide data.

## References

[1] UNEP (United Nations Environment Programme), 2020. Emissions Gap Report 2020.Nairobi.https://www.unep.org/emissions-gap-report-2020

[2] Liang S, Lin X, Liu X (2022). The pathway to China’s carbon neutrality based on an endogenous technology CGE model. Int. J. Environ. Res. Public Health.

[3] Wang H, Zhang Z (2022). Forecasting CO_2_ emissions using a novel grey bernoulli model: A case of Shaanxi province in China. Int. J. Environ. Res. Public Health.

[4] Lu Y, Jiahua P (2013). Disaggregation of carbon emission drivers in Kaya identity and its limitations with regard to policy implications. Adv. Clim. Chang. Res..

[5] Su B, Ang BW (2016). Multi-region comparisons of emission performance: The structural decomposition analysis approach. Ecol. Ind..

[6] Fan F, Lei Y (2017). Factor analysis of energy-related carbon emissions: A case study of Beijing. J. Clean. Prod..

[7] Ang BW (2004). Decomposition analysis for policymaking in energy: Which is the preferred method. Energy Policy.

[8] Kong H, Shi L, Da D (2022). Simulation of China’s carbon emission based on influencing factors. Energies.

[9] Xu SC, He ZX, Long RY (2016). Comparative analysis of the regional contributions to carbon emissions in China. J. Clean. Prod..

[10] Zhang H, Chen B, Deng H (2022). Analysis on the evolution law and influencing factors of Beijing’s power generation carbon emissions. Energy Rep..

[11] Zhao Y, Ding H, Lin X (2021). Carbon emissions peak in the road and marine transportation sectors in view of cost-benefit analysis: A case of Guangdong Province in China. Front. Environ. Sci..

[12] Tian L, Ding Z, Wang Y (2016). Analysis of the driving factors and contributions to carbon emissions of energy consumption from the perspective of the peak volume and time based on LEAP. Sustainability.

[13] Duan H, Dong X, Xie P (2022). Peaking industrial CO_2_ emission in a typical heavy industrial region: From multi-industry and multi-energy type perspectives. Int. J. Environ. Res. Public Health.

[14] Liu D, Xiao B (2018). Can China achieve its carbon emission peaking? A scenario analysis based on STIRPAT and system dynamics model. Ecol. Ind..

[15] Zhao H, Hu J, Hao F (2022). Determinants of Carbon dioxide emissions and their peaking prospect: Evidence from China. Front. Environ. Sci..

[16] Yang Z, Xing Q, Zhang M (2022). Evaluation of the carbon peak in Hebei province based on EES element-STIRPAT model. Fresenius Environ. Bull..

[17] Zhao G, Yu B, An R (2021). Energy system transformations and carbon emission mitigation for China to achieve global 2 C climate target. J. Environ. Manag..

[18] Qin J, Tao H, Zhan M (2019). Scenario analysis of carbon emissions in the energy base, Xinjiang Autonomous Region, China. Sustainability.

[19] Wu CB, Huang GH, Xin BG (2018). Scenario analysis of carbon emissions’ anti-driving effect on Qingdao’s energy structure adjustment with an optimization model, Part I: Carbon emissions peak value prediction. J. Clean. Prod..

[20] Sun LL, Cui HJ, Ge QS (2022). Will China achieve its 2060 carbon neutral commitment from the provincial perspective?. Adv. Clim. Change Res..

[21] Fang K, Li C, Tang Y (2022). China’s pathways to peak carbon emissions: New insights from various industrial sectors. Appl. Energy.

[22] Ma X, Han M, Luo J (2021). The empirical decomposition and peak path of China’s tourism carbon emissions. Environ. Sci. Pollut. Res..

[23] Liu L, Wang K, Wang S (2019). Exploring the driving forces and reduction potential of industrial energy-related CO_2_ emissions during 2001–2030: A case study for Henan Province, China. Sustainability.

[24] Xie Y, Liu X, Chen Q (2020). An integrated assessment for achieving the 2 C target pathway in China by 2030. J. Clean. Prod..

[25] Tian X, Yu Z, Sarkis J (2022). Environmental and resource impacts from an aggressive regionalized carbon peak policy. Environ. Sci. Technol..

[26] Dong F, Qin C, Zhang X (2021). Towards carbon neutrality: The impact of renewable energy development on carbon emission efficiency. Int. J. Environ. Res. Public Health.

[27] Zhang H, Zhang X, Yuan J (2021). Driving forces of carbon emissions in China: A provincial analysis. Environ. Sci. Pollut. Res..

[28] Kaya, Y. Impact of carbon dioxide emission control on GNP growth: interpretation of proposed scenarios. Intergovernmental Panel on Climate Change/Response Strategies Working Group, May, (1989).

[29] Kaya Y, Yokobori K (1997). Environment, Energy, and Economy: Strategies for Sustainability.

[30] Holdren JP, Ehrlich PR (1974). Human population and the global environment: Population growth, rising per capita material consumption, and disruptive technologies have made civilization a global ecological force. Am. Sci..

[31] York R, Rosa EA, Dietz T (2003). STIRPAT, IPAT and ImPACT: analytic tools for unpacking the driving forces of environmental impacts. Ecol. Econ..

[32] Shuai S, Meiting F, Lili Y (2022). Economic restructuring, green technology progress and low-carbon transformation in China: An empirical study from the perspective of overall technology frontier and spatial spillover effect. Manag. World.

[33] Yang X, Li N, Mu H (2022). Population aging, renewable energy budgets and environmental sustainability: does health expenditures matter?. Gondwana Res..

[34] Li H, Yi J, Zhang J (2011). Estimating the effect of the one-child policy on the sex ratio imbalance in China: Identification based on the difference-in-differences. Demography.

[35] Millot A, Maïzi N (2021). From open-loop energy revolutions to closed-loop transition: What drives carbon neutrality?. Technol. Forecast. Soc. Change.

[36] Zheng Y, Xiao JZ, Huang F (2022). How do resource dependence and technological progress affect carbon emissions reduction effect of industrial structure transformation? Empirical research based on the rebound effect in China. Environ. Sci. Pollut. Res..

[37] Hoerl AE, Kennard RW (1970). Ridge regression: Applications to nonorthogonal problems. Technometrics.

